# Superficial Penile Vein Thrombophlebitis After Microsurgical Varicocelectomy: Case Series

**DOI:** 10.7759/cureus.20580

**Published:** 2021-12-21

**Authors:** Moshe Wald, Fiona Armstrong-Pavlik

**Affiliations:** 1 Urology, University of Iowa Hospitals and Clinics, Iowa City, USA; 2 Urology, University of Iowa, Iowa City, USA

**Keywords:** varicocele, aspirin, microsurgical varicocelectomy, superficial penile vein, thrombophlebitis

## Abstract

Varicocele is a common condition, estimated to be present in 15% of the general male population. It consists of abnormal dilation and tortuosity of the internal spermatic veins within the pampiniform plexus of the spermatic cord. In adults, varicocele repair may be considered in cases of scrotal pain, or of infertility associated with impaired sperm parameters. Microsurgical varicocelectomy is currently a common method for varicocele repair.

Superficial penile vein thrombophlebitis has been previously reported to occur after microsurgical varicocelectomy but has not been well recognized as a possible complication of this surgery.

We present three cases of superficial penile vein thrombophlebitis after microsurgical varicocelectomy. Diagnosis on this condition was based on physical examination, mainly a palpable cord-like structure along the involved vein, without overlying redness. Signs and symptoms of superficial penile vein thrombophlebitis resolved after a 4-8-week course of aspirin at the dose of 325 mg daily.

Awareness of this possible post-operative complication and its possible management could be helpful to the clinicians involved in the care of patients with varicoceles.

## Introduction

Varicocele is an abnormal dilation and tortuosity of the internal spermatic veins within the pampiniform plexus of the spermatic cord. Varicocele is a common condition, estimated to be present in 15% of the general male population.

Varicocele has been reported to be possibly associated with scrotal pain [1,2], as well as with impairment in sperm parameters and infertility [3,4]. Thus, varicocele repair may be considered in cases of scrotal pain and infertility associated with impaired sperm parameters. Varicocele repair may be performed via a variety of surgical interventions, including microsurgical or non-microsurgical open procedures through inguinal or subinguinal approaches, laparoscopy, and robotically assisted procedures [5]. Varicocele embolization by Interventional Radiology has also been reported as a possible method for varicocele repair [6].

Microsurgical varicocelectomy is currently a common method for varicocele repair. This procedure involves exposure of a segment of the spermatic cord through an inguinal or subinguinal approach, followed by the positioning of an operating microscope over the surgical field, allowing for isolation and subsequent ligation of the dilated veins within the spermatic cord under optical magnification, while sparing the testicular artery and lymphatic channels.

Possible complications of microsurgical varicocelectomy include hydrocele formation, testicular artery injury, and varicocele recurrence [5]. Hydrocele formation after varicocelectomy has been attributed to the obstruction of lymphatic channels within the spermatic cord [7]. Hydrocele formation is the most common complication reported after non-microsurgical varicocele repair, with an average incidence of approximately 7%. However, the use of optical magnification enables the identification and sparing of lymphatic channels, and has been reported to almost eliminate the risk of hydrocele formation after varicocele repair [8-10]. Testicular artery injury has been associated with the risks of testicular atrophy and impaired spermatogenesis. These risks could be minimized by the use of optical magnification and an intra-operative Doppler device, which improve the identification and preservation of the testicular artery [5]. Microsurgical varicocelectomy with delivery of the testis has been reported to lower the incidence of varicocele recurrence to less than 1%, as opposed to 9% using conventional inguinal techniques [8,9].

While a single series published in 2011 reported superficial penile vein thrombophlebitis after subinguinal varicocelectomy [11], this condition has not been well recognized as a possible complication of this surgery.

We present three cases of superficial penile vein thrombophlebitis after microsurgical varicocelectomy.

## Case presentation

Case 1

A 35-year-old male presented with a history of left varicocele associated with left-sided scrotal pain. He underwent a left microsurgical varicocelectomy in October 2016. At his one-month follow-up, he reported complete resolution of the left-sided scrotal pain that he was experiencing for several years pre-operatively. He did feel, however, that the left testicle is somewhat more tender to the touch than before. He also noted some increased prominence of a superficial vein of the penis, with some discomfort in that area with erections. Erections were otherwise normal. Genital exam revealed both testicles to be normal in consistency without palpable masses, mild tenderness to palpation along the left epididymis without associated swelling or skin changes. There was a palpable thin cord within the superficial dorsal vein of the penis without associated erythema or tenderness. A surgical incision in the left groin was well healed. He was then prescribed a 10-day course of ciprofloxacin 500 mg BID, as well as a one-month course of aspirin 325 mg daily.

Three weeks later, he reported that the penile vein prominence was less prominent than before, but still felt slightly more pronounced than at baseline, with some persistent mild discomfort with erections. He denied any difficulty with urination. On exam, the previously noted cord within the superficial vein of the penis has almost completely resolved and was barely palpable, with no appreciable tenderness to palpation along with the testicle or epididymitis bilaterally. He was instructed to complete a month’s course of aspirin and return to the clinic on an as needed basis.

Case 2

A 22-year-old man presented to our clinic with left varicocele, oligospermia, and history of microscopic hematuria. He underwent left microsurgical varicocelectomy and cystoscopy in December 2017. Cystoscopy was unremarkable. He called after surgery and reported a thin cord-like structure felt superficially at the base of the penis. He was started on a course of daily aspirin. At a one-month follow-up, he reported that this has been slowly improving with aspirin. On exam, there was a thin cord-like structure felt superficially at the base of the penis, suggestive of superficial thrombophlebitis. He was then instructed to continue aspirin for one month.

At his follow-up four months post-operatively, his penile symptoms had resolved, and he had no hematuria. On exam, there were no varicocele and no evidence for superficial penile vein thrombophlebitis. Semen analysis showed sperm concentration of 11.9 million/mL and motility of 54.6%, improved from pre-operative 6.5 million/mL and 29.2%, respectively. The patient was about to graduate and move elsewhere; he was advised to have urinalysis and semen analysis in one year at his new location.

Case 3

A 32-year-old gentleman presented with a history of right laparoscopic inguinal hernia repair in 2016 at which time he was noted to have a large left varicocele. The varicocele was not associated with any scrotal pain. He presented to our clinic for evaluation of primary infertility. He and his wife have been attempting to conceive for 15 months with no success. Pre-operative semen analysis showed low sperm concentration and motility at 1.8 million/mL and 2.9%, respectively. He underwent a left microsurgical varicocelectomy in December 2018.

One month after surgery, he reported that he noticed a hard vein on the dorsal shaft of the penis, without any pain or difficulty with erections. Physical exam revealed a hard superficial vein on the dorsal penile shaft with no signs of infection. He was placed on a six-week course of aspirin.

Four months after surgery, he reported that the hard vein on the dorsal penile shaft has completely resolved. Semen analysis showed sperm concentration of 9.8 million/mL and motility of 20.6%, improved from pre-operative 1.8 million/mL and 2.9%, respectively. The patient and his wife elected to attempt conception naturally and follow up in six months if no pregnancy is achieved by that time.

## Discussion

The notable incidence of varicocele and its possible association with fertility impairment and scrotal pain makes varicocele repair a fairly common intervention, especially in younger men. While various methods for varicocele repair exist, microsurgical varicocelectomy is currently a common method for varicocele repair, particularly in the setting of infertility. Possible complications of this surgery that involve the genital organs could be concerning to men who are seeking fertility, especially if perceived as having the potential to compromise sexual function.

We present three patients who developed superficial penile vein thrombophlebitis following microsurgical varicocelectomy. The diagnosis of superficial penile vein thrombophlebitis after microsurgical varicocelectomy was based on the constellation of symptoms and physical examination findings. The mainstay of the latter was a palpable cord-like structure along the involved vein, without overlying redness and with no interruption of erectile function. In the absence of clinical evidence for a superimposed infection, these three patients were treated with a course of aspirin at the dose of 325 mg daily. The length of such a course varied from four to eight weeks, depending on the extent and duration of symptoms. In all three patients, signs and symptoms of superficial penile vein thrombophlebitis resolved after a course of aspirin.

A single series of superficial dorsal penile vein thrombosis following subinguinal varicocelectomy was published by Arango et al in 2011 [11]. In this series, superficial dorsal penile vein thrombosis occurred in 2.1% of 326 patients who underwent this surgery. The three patients included in our series of superficial dorsal penile vein thrombosis after microsurgical varicocelectomy were identified among 406 patients who underwent this surgery, suggesting a lower incidence of this post-operative complication.

The mechanism for the development of superficial penile vein thrombophlebitis or thrombosis after microsurgical varicocelectomy is not clear, especially as this particular procedure involves the ligation of dilated veins within the spermatic cord at the subinguinal or inguinal level, without any obvious involvement of the penile vessels. It is possible that the vein ligation at the time of microsurgical varicocelectomy, perhaps more at the early post-operative period, causes alteration in testicular venous drainage, which in turn could transiently affect adjacent venous systems. Further studies of the underlying mechanisms for the development of this type of post-operative thrombophlebitis are needed and could possibly offer ways to avoid this complication.

Mondor's disease (MD) is a rare condition that manifests with a palpable cord-like induration on the body surface. MD involving the anterolateral thoracoabdominal wall has been generally considered as original MD. Penile MD (PMD) is recognized as a variant of MD. The vast majority of MD cases were reported to be thrombophlebitis of a superficial vein, although some were reported to represent lymphangitis or a combination of both etiologies. Reported risk factors for PMD include vessel-wall damage (related to vigorous sexual activity, penile trauma, or use of vacuum erection device), blood stasis (such as prolonged erections), and conditions of hypercoagulation [12]. 

Of note, none of the three patients included in our case series had a history of clotting disorders, hypertension, diabetes mellitus, a history of thrombophlebitis elsewhere, or any of the above risk factors for PMD. The lack of such risk factors could possibly support the assumption that this type of thrombophlebitis or thrombosis is related to a transient impact of the alteration in testicular venous drainage following varicocelectomy, rather than to an underlying systemic condition.

Our case series presents three patients who developed superficial penile vein thrombophlebitis after microsurgical varicocelectomy. This condition was previously reported but has not been well recognized as a possible complication of this surgery. Awareness of this possible post-operative complication, as well as its natural history and possible management, could be helpful to the clinicians involved in the care of patients with varicoceles.

## Conclusions

Clotting in superficial penile veins following microsurgical varicocelectomy is a rare occurrence.

Our case series suggests the effectiveness of a course of aspirin in the management of this condition. However, further research is needed to better understand the mechanism and risk factors for thrombophlebitis in this region as well as the best way to treat it.
